# Factors Determining the Southern Range Limit of *Hemigrapsus sanguineus* in the Western Atlantic

**DOI:** 10.1002/ece3.71518

**Published:** 2025-06-03

**Authors:** Luke Ashworth, Margo Harris, David L. Neu, Blaine D. Griffen

**Affiliations:** ^1^ Department of Biology Brigham Young University Provo Utah USA

**Keywords:** biogeographic barrier, habitat suitability, invasive biology, marine invasions, marine invertebrate, nearshore

## Abstract

The southern range limit of the invasive Asian shore crab, *Hemigrapsus sanguineus,* along the United States East coast is further north than expected based on its native distribution. We investigated potential factors that may limit the southward spread of this species along the Mid‐Atlantic and South Atlantic bights from Virginia to South Carolina, including metabolic constraints, food availability, and habitat limitation. We searched sites identified as potential habitat for *H. sanguineus* to verify the presence/absence of the crab, measured the metabolic rates of crabs at their current southern range edge for comparison with previous measurements made further north on the New Hampshire coast, used digital images captured at each site to determine whether the availability of potential food decreases south of the current range limit, and used Google Earth to measure distances between suitable habitat patches north and south of the current range limit to determine whether habitat availability limits the range expansion toward the south. We encountered the species ~64 km further south than the documented range limit at Oregon Inlet, North Carolina. We found no difference in metabolism between crabs at the southern range edge compared to crabs from New Hampshire, and no consistent difference in the abundance of available food between sites north and south of the current range limit. However, we found greater distances between suitable hard‐substrate sites south of the current range limit than between sites found within the current range. We suggest that the availability of suitable habitat is the primary driver limiting the further southward range expansion of *H. sanguineus* .

## Graphical abstract

We examined the southern range limit of the invasive Asian shore crab Hemigrapsus sanguinues. We show that the most likely determinant of the southern range edge is the availability of suitable habitat on the open coast.

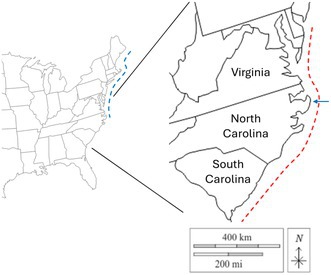

## 1 Introduction

Nearshore marine communities have a long history of anthropogenically facilitated invasions of non‐native species. For instance, Ens et al. (2022), in their review of the invasive impacts of *Carcinus maenas*, identified its long track record of invading nearshore communities across the globe and later becoming the dominant species among the resident competitors. Historically successful invasive species can be expected to continue to expand their ranges as modern advances in transportation and shipping intensify the global exchange of goods and organisms.

Biogeographic barriers play an important role in limiting range expansions. For instance, along the east coast of North America, biogeographic boundaries occur at Cape Hatteras, Cape Cod, and the Bay of Fundy, and are generally created by interactions between ocean currents, the depth distribution of organisms, and the duration of pelagic larval dispersal (Pappalardo et al. 2015). However, anthropogenic habitat modifications can enable species to expand their ranges across natural biogeographic boundaries. For example, several native species of bivalve previously limited to the southern coast of China have recently spread northward past the Yangtze River, a historic species boundary, as concrete and other structures have been built in areas previously without hard substrate (Wang et al. 2020). Similarly, the construction of boat docks in the southeastern United States has facilitated the range expansion of mangrove tree crabs into saltmarshes that occur north of their historic mangrove‐associated range limit (Cannizzo and Griffen 2019).

The invasive spread of the tropical reef dwelling fish *Chromis limbata* demonstrates the role of isolated “islands” of suitable habitat in determining the range limits of a newly introduced species. Researchers monitoring the spread of the fish have identified healthy populations in reefs in cooler waters along Brazil's southern coast, as well as populations in warmer waters to the north inhabiting deeper, and thus colder, reefs. These deep‐water reefs appear to allow the spread and recruitment of *C. limbata* into northern waters beyond its generally accepted climate tolerances (Anderson et al. 2020).

Habitat suitability, and by extension the range limits, for an invading species cannot always be tied to the realized niche in their native ranges. For instance, the spread and realized niche of the invasive common lionfish ( *Pterois miles* ) in the Mediterranean Sea are determined by parameters that are distinct compared with its native range (Poursanidis et al. 2020). This indicates that habitat suitability in invaded regions may differ from that in native habitats due to differences in species interactions, organismal tolerance, or other factors, making it difficult to determine habitat suitability in invaded regions. Even beyond invasive species, the factors that determine range limits in general are understudied, and the theoretical predictions in this area far outstrip empirical tests of those theories (Sexton et al. 2009).

The Asian shore crab *Hemigrapsus sanguineus* is native to the western Pacific, occurring in Hong Kong, Taiwan, China, Korea, and Russia (~20° to 50° N latitude as summarized in McDermott 1998), but has proven to be a successful invader in both the eastern and western North Atlantic (Epifanio 2013; Dauvin and Dufossé 2011). In its native range, *H. sanguineus* occurs most abundantly in the upper‐ and mid‐intertidal zones of rocky shores on open coasts and in lower reaches of estuaries where it takes refuge under loose stones that overlay the substrate (Lohrer, Whitlatch, et al. 2000). Consequently, *H. sanguineus* densities increase with increasing boulder coverage on the shore (Lohrer, Whitlatch, et al. 2000). Furthermore, the experimental removal of rock structure at *H. sanguineus'* preferred tidal height in its native range in Tanabe Bay, Japan, resulted in a significant decrease in abundance (Lohrer, Fukui, et al. 2000). *H. sanguineus* is omnivorous, with approximately 50% of its diet in the native range coming from plants, while the rest is a mix of detritus and common invertebrates, including bivalves, gastropods, arthropods, and polychaetes (Lohrer, Whitlatch, et al. 2000).

**FIGURE 1:**
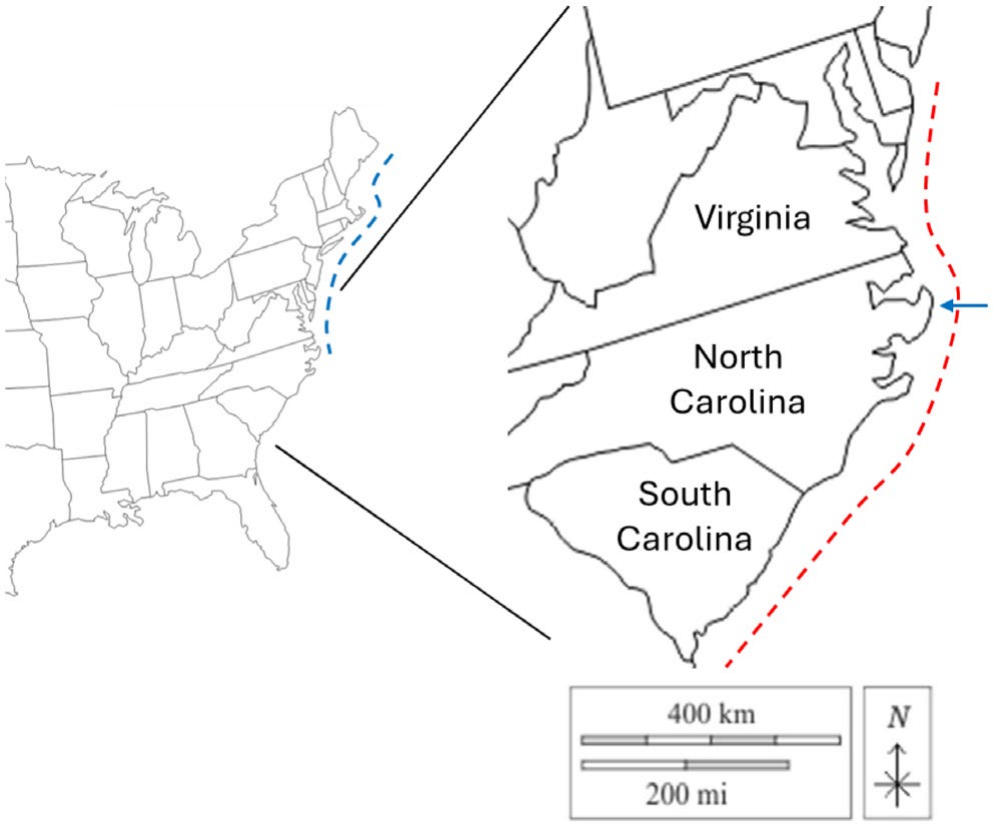
Map of study region. Left part of figure shows full invaded range of *Hemigrapsus sanguineus* along the United States east coast (blue dashed line). Right part of figure shows expansion of region examined in this study, including the region examined for hard substrate availability (red dashed line) and the current southern range limit of *H. sanguineus* at Oregon Inlet, North Carolina (blue arrow).

*H. sanguineus* was first documented in the western Atlantic at Townsend Inlet, New Jersey in 1988 (McDermott 1991). Since then, it has become one of the most wide‐ranging species in rocky intertidal environments. Based on genetic markers, Blakeslee et al. (2017) conclude that *H. sanguineus* was introduced to the United States from Japan, most likely through multiple introduction events via ballast water. By 1993, the species had spread from Woods Hole, MA to Chesapeake Bay (McDermott 1995), and by 1995, the species had reached its currently documented southern range limit at Oregon Inlet on the outer banks of North Carolina (McDermott 1998). This southern range limit at roughly 35° N (Figure 1) is much more restricted than the native range that extends to ~20° N, level with Cuba in the Western Hemisphere. In contrast to this, *H. sanguineus* has continued to expand its northern range limit, reaching mid‐coast Maine by 2007 (Griffen and Delaney 2007), and recent work has shown the presence of a breeding population of *H. sanguineus* in southern Canada (Ramey‐Balci 2023).

In its invaded range, *H. sanguineus* appears to have expanded its habitat niche somewhat. For instance, Brousseau et al. (2003) observed *H. sanguineus* inhabiting burrows constructed by the mud fiddler crab *Uca pugnax* on the edge of a salt marsh. Furthermore, while it was never observed subtidally in its native range (Lohrer, Whitlatch, et al. 2000), Gilman and Grace (2009) report finding *H. sanguineus* subtidally to a depth of 3 m in Connecticut, though this appears to occur in winter and may be a strategy to avoid freezing temperatures in the intertidal zone. Despite these notable exceptions, *H. sanguineus* in the invaded range fills much the same habitat and trophic niche as it fills in its native range. Specifically, it is found in coastal and lower estuarine rocky intertidal habitats, where its abundance increases with loose rocks sitting atop the substrate, and it subsists on an omnivorous diet of algae, detritus, bivalves, crustaceans, and other invertebrates (Lohrer, Whitlatch, et al. 2000; Ledesma and O'Connor 2001; Griffen et al. 2008). Additionally, as in the native range (Lohrer, Fukui, et al. 2000), *H. sanguineus* in the invaded range shows a strong preference for hiding under shelter (Steinberg and Epifanio 2011). Rock refuges could potentially provide multiple benefits. Predation on *H. sanguineus* occurs during both high and low‐tide periods, and refuge habitats reduce predation risk (Montanaro and O'Connor 2024). In addition, rocks in intertidal regions ameliorate low‐tide temperature extremes, providing thermal refuges for intertidal organisms (Gunderson et al. 2019). No data on thermal tolerance for *H. sanguineus* are available in the peer‐reviewed published literature; however, unpublished data from an undergraduate research project suggest that thermal tolerances may be reached around 37°C (Rubock 2020).

Since its arrival in the western Atlantic, *H. sanguineus h*as significantly impacted rocky intertidal ecosystems throughout New England and the Mid‐Atlantic, displacing native fiddler and mud crabs as well as heavily predating on a wide range of species such as littorine snails, mussels, barnacles, and crustaceans, including the economically valued Maine lobster. (Kraemer et al. 2007; Lord and Williams 2017). Additionally, *H. sanguineus* appears to have the upper hand in competitive interactions for habitat and food with the previously established invasive European green crab *Carcinus maenas* that occupies the same rocky intertidal habitats (Jensen et al. 2002). Consequently, it remains unclear why the southern range limit of *H. sanguineus* is truncated compared to its native southern range limit, while the northern invasive range limit continues to expand.

Multiple factors could conceivably contribute to *H. sanguineus* ' truncated southern range limit. First, rocky shores characteristic of New England that are attributed to historic glacial activity are not shared with the southern coastline, and it has been hypothesized that the lack of rocky substrate south of Cape Hatteras may limit colonization of *H. sanguineus* further south (McDermott 1998; Epifanio 2013). Second, clutch sizes of gravid females are smaller at the southern range edge than throughout the rest of the range (Griffen et al. 2024), and this could reflect reductions in energy intake. While diet quality does not appear to vary substantially throughout the invasive range (Reese et al. 2023), reduced fecundity at the southern range edge could reflect reduced food availability. Third, reduced fecundity could alternatively reflect increased energetic costs at the southern range edge due to higher temperatures. Jungblut et al. (2018), studying invasive populations in the eastern Atlantic, showed that the metabolism of *H. sanguineus* is highly temperature dependent, with energetic costs being two times higher than for *C. maenas* in warmer temperatures, despite the two species having similar metabolic rates at cooler temperatures. However, as noted by Epifanio (2013), the native and western Atlantic invasive ranges are both influenced by western boundary currents and have similar ocean temperatures when comparing comparable latitudes. Thus, low‐tide air temperatures may be more critical than ocean temperatures.

We tested the hypothesis that the southern range limit of *H. sanguineus* in the western Atlantic remains at Oregon Inlet, North Carolina, as described by Epifanio (2013). We additionally tested the hypothesis that the southern range limit is influenced by one or more of three factors: first, the availability of and distance between suitable hard‐substrate habitats; second, the availability of suitable foods for this generalist consumer; and third, increased metabolic costs during low tide due to higher air temperatures at the southern range edge.

## 2 Methods

### 2.1 *H. sanguineus* Presence North and South of Its Documented Range Limit

We identified 11 sites with extensive intertidal hard substrate (man‐made rock piles creating harbor entrances, groins, riprap) that could potentially serve as suitable habitat for *H. sanguineus* . Four of these sites occurred north of the documented range limit, one was at the documented range limit (Oregon Inlet, North Carolina), and six occurred south of this range limit (Table 1). We visited each site at low tide between June 12 and June 23, 2023, and searched exhaustively for 2 h (5 people × 2 h = 10 person h at each site) by overturning rocks and boulders, searching crevices, etc. In addition, we searched all pilings and rock walls when present. We collected all *H. sanguineus* that were found. This region is dominated by sandy shores and salt marshes, and most locations within this region are not suitable for *H. sanguineus* . The sampled sites were selected because of the relatively large amount of hard substrate at each, and the nature of this hard substrate in providing refuge that crabs could hide underneath, providing the greatest likelihood of finding *H. sanguineus* .

**TABLE 1:** *Hemigrapsus sanguineus* sampling sites by name and GPS location.

Site	Approximate GPS location
Hampton Virginia, Buckroe Beach	37°02′46″ N 76°17′11″ W
Hampton Virginia, Buckroe Beach	37°03′42″ N 76°16′52″ W
Norfolk Virginia, East Beach	36°55′47″ N 76°10′49″ W
Corolla North Carolina, Whalehead Bay	36°20′58″ N 75°49′41″ W
Oregon Inlet, North Carolina	35°46′03″ N 75°31′33″ W
Cape Hatteras North Carolina, Teach's Marina	35°12′40″ N 75°42′04″ W
Cape Hatteras North Carolina, Coast Guard Base	35°12′31″ N 75°42′15″ W
Cedar Island Ferry, North Carolina	35°01′10″ N 76°18′52″ W
Wilmington, North Carolina, Fort Fisher	33°58′20″ N 77°54′53″ W
Murrells Inlet, South Jetty, South Carolina	33°31′36″ N 79°01′52″ W
Folly Beach, South Carolina	32°38′23″ N 79°58′01″ W

### 2.2 Hard‐Substrate Availability

As noted above, *H. sanguineus* prefers to inhabit intertidal habitats with abundant boulders, cobble, or other loose rock sitting atop the substrate. This habitat provides structures under which crabs can take refuge. By contrast, *H. sanguineus* is not generally found on bare substrate, on rock walls that lack an algal canopy, or on pilings, boat docks, or other such structures. We therefore focused our search on intertidal regions that had boulders or other hard structures that could provide the required refuge, focusing on the average distance between potentially suitable habitats.

We used Google Earth to view images of the coastline from the Maryland‐Delaware border (38°27′04″ N 75°02′57″ W) to the South Carolina‐Georgia border (32°02′19″ N 80°50′33″ W) (Figure 1). At a ~ 0.5 km viewing scale, we surveyed the coastline including both open coastal areas and areas within bays and estuaries, such as the Pamlico‐Albemarle region, North Carolina, and marked each visible rocky structure. For bays and estuaries, we included areas within 15 km of the nearest connection to the open ocean. Satellite images used in Google Earth may be taken at any point in the tidal cycle. Therefore, to ensure that we did not miss lower intertidal structures that would be covered at high tide, we used the Historical Imagery feature of Google Earth to visually identify recent images taken during low‐tide periods (determined by the relative width of the beach and darkened color of recently exposed sand across images). We then measured and recorded the distance between the nearest edge of each marked site to the nearest edge of the next marked site using the measure function on Google Earth. In determining these distances, we followed the natural contours of the coastline rather than measuring a straight line between adjacent sites. We recorded the coordinates of each site and noted whether the site was along the primary ocean shoreline or in a bay. We acknowledge that these methods are unlikely to identify every possible suitable structure that could be inhabited by *H. sanguineus* . However, this reflected the feasibility in examining such a large range of coastline.

We conducted a two‐way ANOVA with the log distance between suitable hard‐substrate sites as the response variable, and with inside/outside the current *H. sanguineus* range as one factor and with bay/ocean (for the Outer Banks, Albermarle‐Pamlico region of North Carolina) as a second factor. Bays and estuaries that occurred south of the Outer Banks region (i.e., south of the Albermarle‐Pamlico region) were also included in our analysis; however, because they are not interconnected in a way that larval recruits should be expected to move between bays without spending time in open coastal waters, bays south of the Outer Banks were included in the factor “ocean” for purposes of determining distances between hard substrates (i.e., the distance larvae would need to travel between adjacent sites). There was a significant interaction term between the two predictor variables, but neither main effect was significant. For clarity of interpretation, we therefore conducted separate one‐way ANOVAs for the open coast and for bays and using only inside/outside the current *H. sanguineus* range as the predictor variable.

One function provided by a suitable hard substrate is to reduce the temperature to which crabs are exposed. Therefore, in addition to examining the availability of hard substrates, we also investigated the role of boulder size in ameliorating temperature extremes during low‐tide summertime periods on shores where *H. sanguineus* occurs. In the mid‐ to upper‐intertidal zone at Odiorne Point State Park, New Hampshire, we measured temperatures under boulders in July 2022 between 3 to 5 pm at low tide. Maximum observed temperature on the (uncovered) substrate surface during sampling was 46.5°C. We flipped haphazardly chosen boulders of a range of sizes on a southward‐facing shore and, using a Flir C2 camera, we immediately took a thermal image of the substrate under each boulder. We then measured the maximum thickness of the rock to the nearest centimeter. We determined the minimum temperature of the substrate on each thermal image using Flir Tools. We then used nonlinear regression, fitting the equation Temp = *a* × Thickness^*b*^, to determine how temperature changed under boulders as a function of boulder thickness.

### 2.3 Food Availability

At each site listed in Table 1, we nonrandomly selected 10 quadrat (1 m^2^) locations at low tide in an attempt to record the types of food present at the site. Given the omnivorous diet of *H. sanguineus* , we included all macroalgae and invertebrates as potential prey in our analysis. Quadrats were located on rock faces and crevices, usually on large boulders that comprised the limited hard substrate at the site. Quadrats were selectively positioned to include areas of abundant potential prey to facilitate our goal of determining whether acceptable food was available at a site (as opposed to quantifying prey abundance using random sampling). We then took a digital picture of each quadrat. When boulders were not embedded in the sand and could be overturned (i.e., when *H. sanguineus* could take refuge under them), we took photographs of the underside sides of boulders as well. We analyzed photos using 100 evenly spaced intersection points, digitally placed within each quadrat to determine the percent coverage of algae. Animal species were counted individually within the images of the quadrats. We then conducted an analysis of similarity using Bray–Curtis distance (results were qualitatively identical when a Manhattan or Euclidean distance were used), comparing sites within the current known range of *H. sanguineus* and those south of the range limit. In addition to analyzing the potential prey community as a whole, we also compared the density of each potential prey individually inside and outside the current range using *t*‐tests. Given our nonrandom sampling approach, these should be thought of as maximum densities. It should be noted that the limited number of sampling sites and relatively high variation in prey abundances yield low power for detecting significant differences. However, our goal was not necessarily to demonstrate statistical differences, but was instead to simply determine whether appropriate food types were available at sites outside the current invaded range.

### 2.4 Metabolic Rates

Metabolic rate may be influenced by a wide range of factors, including body size, temperature, body condition, reproductive state, food consumption, and activity level. Our goal was to compare metabolic rates of crabs at the southern range edge to crabs that were as similar as possible, but that occur further north, to assess whether crabs at the southern range edge differed in their energy expenditure from conspecifics throughout the rest of the invasive range.

We collected 19 adult *H. sanguineus* individuals (both sexes) from Oregon Inlet, North Carolina, the current known southern limit of the invasive range, on June 13, 2023. We then immediately measured their metabolic rates on the beach while shading the crabs from direct sunlight by placing them under rocks, similar to what they would normally experience during low tide. We replicated the methods used by Fletcher et al. (2022) for measuring oxygen consumption for this species, as follows.

Individual crabs were placed in 150‐ml plastic syringes sealed at the ends with silicone, and with 8‐mm holes drilled into the barrel of each syringe from which gas samples were extracted. We adjusted the volume of each syringe based on the size of the crab. The syringes of individuals with a carapace width of 20 mm or greater were between 70 and 100 mL, and for smaller individuals, we used volumes between 40 and 70 mL. Before sealing the syringe, we recorded barometric pressure, ambient temperature, and relative humidity using a BTMeter (Model 100‐AAP). After placing the crabs into the chambers, we allowed a 5‐min acclimation period before sealing the hole in the syringe barrel using a septum designed for use in headspace gas analysis (Bridge Analyzers Incorporated, model #001620). We recorded start times and end times for each trial in order to calculate the trial duration for each crab. Trials were run for 45–60 min, with smaller crabs being given more time to ensure the changes in oxygen would be measurable. At the conclusion of each trial, we measured the final partial concentration of oxygen in the chamber by inserting a needle into the sampling port connected to a multi‐gas oxygen probe from Forensics Detectors (Model #FD‐600, 0.01% resolution) set to withdraw gas samples at a rate of 0.5 L min^−1^. Each crab was then placed into a separate sample bag and was returned to Brigham Young University (Provo, Utah) on dry ice, where we assessed the size (volume), sex, number of missing limbs, reproductive state (by dissection), and total dry mass of each.

We calculated the metabolic rate using the following methods from Lighton (2018):

$${\text{VolO}}_{2}=\frac{\left[V\left(F_{i}O_{2}-F_{e}O_{2}\right)-F_{e}O_{2}\left(\mathrm{Vol}H_{2}O\right)\right]}{\left[1-F_{e}O_{2}\left(1-\mathrm{RQ}\right)\right]}$$

where VolO_2_ is the volume of oxygen consumed, *V* is the volume of the gas in the chamber, *F*_*i*_ *O*_*2*_ and *F*_*e*_ *O*_*2*_ are the initial and final fractional concentrations of oxygen in the chamber (measured using the gas meter), VolH_2_O is the change in the volume of water vapor in the chamber, and RQ is the respiratory quotient that represents the ratio of CO_2_ produced to O_2_ consumed based on diet. This value ranges from 0.7 to 1.0 (Lighton 2018), and we set it to 0.85 based on the omnivorous nature of *H. sanguineus* and because this middle‐of‐the‐road value minimizes the possible error in metabolic rate at 3% (Vleck 1987). We determined *V* by subtracting the volume of the crab (measured by water displacement in a graduated cylinder during dissection) from the volume of the chamber (which varied based on crab size). We determined VolH_2_O based on the temperature, relative humidity, and barometric pressure using a calculator available at www.respirometry.org.

For each crab, we identified an equivalent crab (same sex, size, reproductive state, number of missing limbs, trial temperatures) from crabs for which we had previously measured metabolic rates using the same methods on the Maine and New Hampshire coasts. Mean temperature during the North Carolina measurements was 23.3°C, while mean temperature during trials in Maine and New Hampshire was 23.8°C. To facilitate comparisons, we standardized the metabolic rate of each crab to standard temperature and pressure using the equation 2.1 from Lighton (2018). There were two outlier datapoints—one from each site. We therefore used a generalized linear model with a gamma distribution to compare the metabolic rates of crabs on the range edge to those further north. To ensure that these outliers did not unduly influence results, we also removed the outliers and repeated the analysis using a general linear model.

## 3 Results

### 3.1 *H. sanguineus* Presence North and South of Its Documented Range Limit

We found *H. sanguineus* at each site within its currently documented range, including at Oregon Inlet, North Carolina. We also found four individuals (one adult male and three juveniles) on rocky riprap and rubble on Coast Guard property near the Cape Hatteras ferry terminal, North Carolina (just to the south of Cape Hatteras and within the Pamlico Sound). This is approximately 88 km or 62 km south of the previously documented range limit at Oregon Inlet, depending on whether the larvae arrived there by following the contours of the open coast or using a straight‐line distance through the Pamlico Sound, respectively. All crabs were found under boulders, and no crabs were found on bare sand, on exposed rock faces, or on pilings or rock walls. We note that no reproductive females were found at this site. It is therefore not clear whether the observed individuals represent part of a sustained population or simply occasional recruits that settle, grow, and die at this location without ever reproducing.

### 3.2 Hard‐Substrate Availability

**FIGURE 2:**
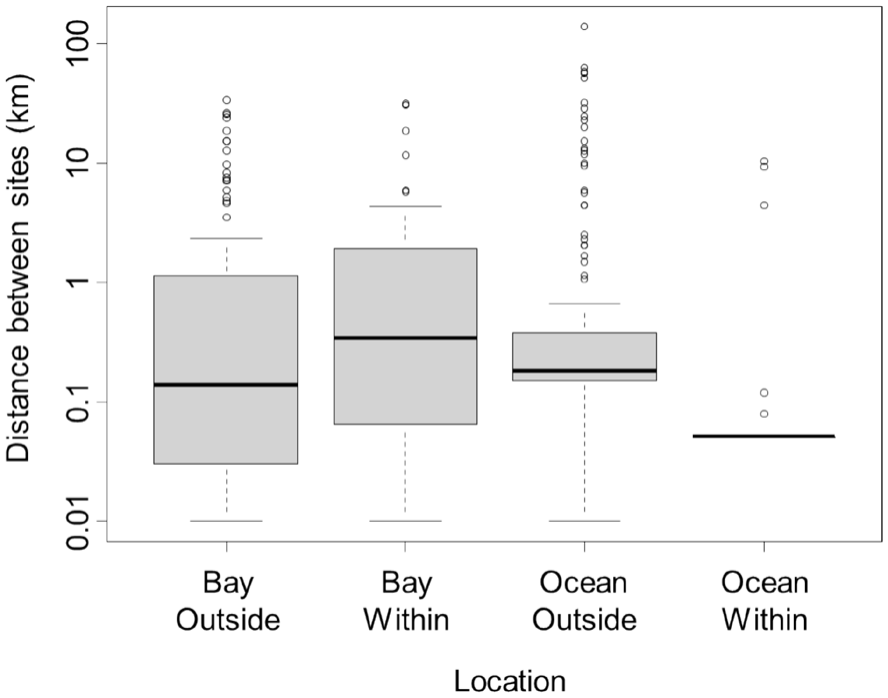
Distance between adjacent suitable hard‐substrate sites north and south of the current range limit of *Hemigrapsus sanguineus*. On the *x*‐axis, “Bay” refers to the Albermarle‐Pamlico region, while “Ocean” refers to both the open ocean along the Outer Banks of North Carolina, but also bays and estuaries to the south of the Albermarle‐Pamlico region that are connected only by open ocean larval transport. “Outside” and “Within” refer to outside and within the current range of *H. sanguineus*. There was a significantly greater distance outside the current range on the open ocean, but no difference between sites within and outside the current range of *H. sanguineus* when comparing within the Albermarle‐Pamlico region. Boxes encompass the interquartile range. Whiskers encompass 1.5× the interquartile range. Circles show datapoints that fall outside this range. Solid line shows median distances.

We evaluated 262 km of coastline within the range of *H. sanguineus* and found 23 hard‐substrate sites, while to the south of *H. sanguineus* ' range we sampled 623 km of open coast and found 121 hard‐substrate sites. In contrast, within bays, we searched approximately 142 km of coastline and found 52 hard‐substrate sites within the range of *H. sanguineus* , and searched 25 km of coastline to the south of *H. sanguineus* ' range and found 23 hard‐substrate sites. Overall, there was a shorter distance between suitable hard‐substrate habitats on the open coast within the current western Atlantic range of *H. sanguineus* than south of the current range limit (ANOVA, *F*_1,142_ = 9.557, *p* = 0.002, Figure 2). The distance between suitable habitats within bays was more variable than on the open coast, but was not statistically different within the current range of *H. sanguineus* compared to south of the current range limit (ANOVA, *F*_1,146_ = 0.559, *p* = 0.456, Figure 2).

**FIGURE 3:**
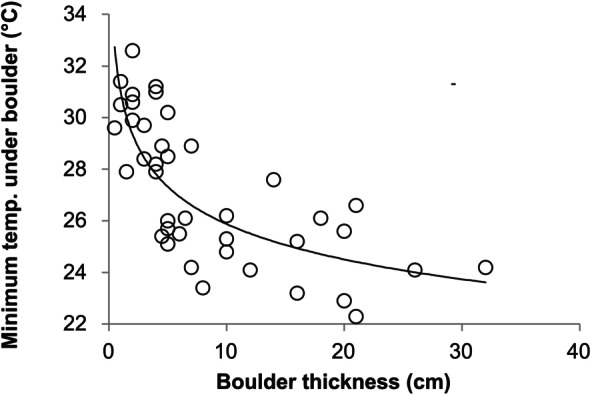
Relationship between boulder thickness and the minimum temperature under the boulder during summer afternoon low tides on the New Hampshire coast.

The presence of large boulders sitting atop the substrate provides a thermal refuge for crabs that is not available with either small cobble or with no shelter at all. Specifically, we found that minimum substrate temperatures under boulders in New Hampshire decreased asymptotically with rock thickness raised to the −0.079 power (*t* = −7.03, *p* < 0.0001, Figure 3).

### 3.3 Food Availability

**FIGURE 4:**
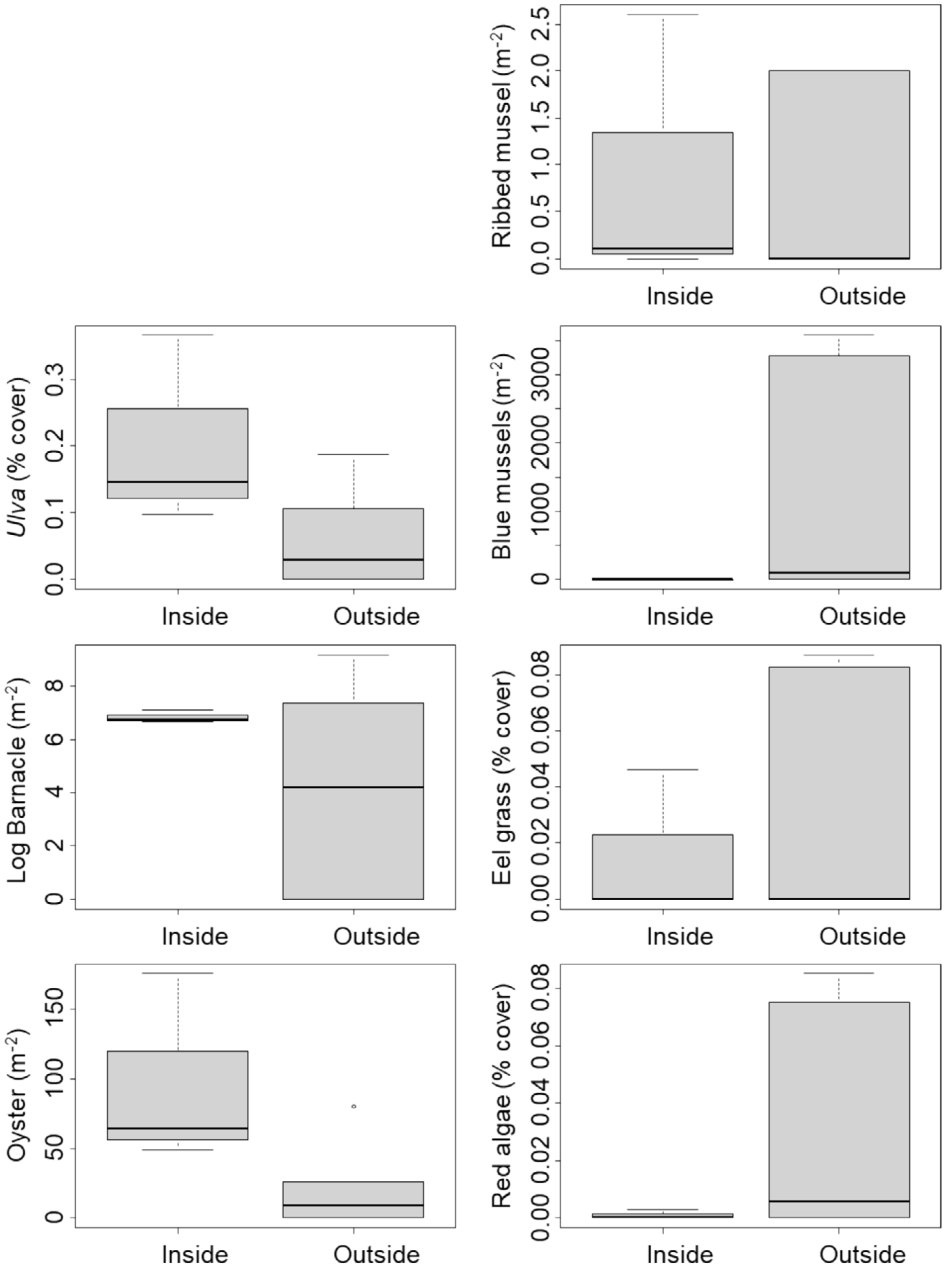
Abundance of potential prey items found at hard‐substrate sites both inside and outside the current range limit of *Hemigrapsus sanguineus* . Boxes are as described in Figure 1.

There were no differences in the community structure of potential prey species at sites inside and outside of the current range of *H. sanguineus* (ANOSIM, *p* = 0.635, Figure 4). When comparing the maximum densities of individual prey species inside vs. outside the current range using *t*‐tests, there were also no differences, including for oysters and *Ulva*, where apparent differences shown in Figure 4 are not significant (all *p* > 0.15).

While there were no clear differences in food availability inside and outside of the current invasive range, the accessibility of that food did change. To the south of the current range, potential food was found primarily on vertical faces of boulders that were embedded in a sandy substrate. The exception to this was large boulders of rock jetties that had deep crevices that extended subtidally (e.g., at Murrels Inlet, South Carolina). These likely included food on horizontal, protected surfaces on the bottoms of boulders, but we were not able to assess this due to the depth and size of the boulders that prohibited their overturning.

### 3.4 Metabolic Rates

**FIGURE 5:**
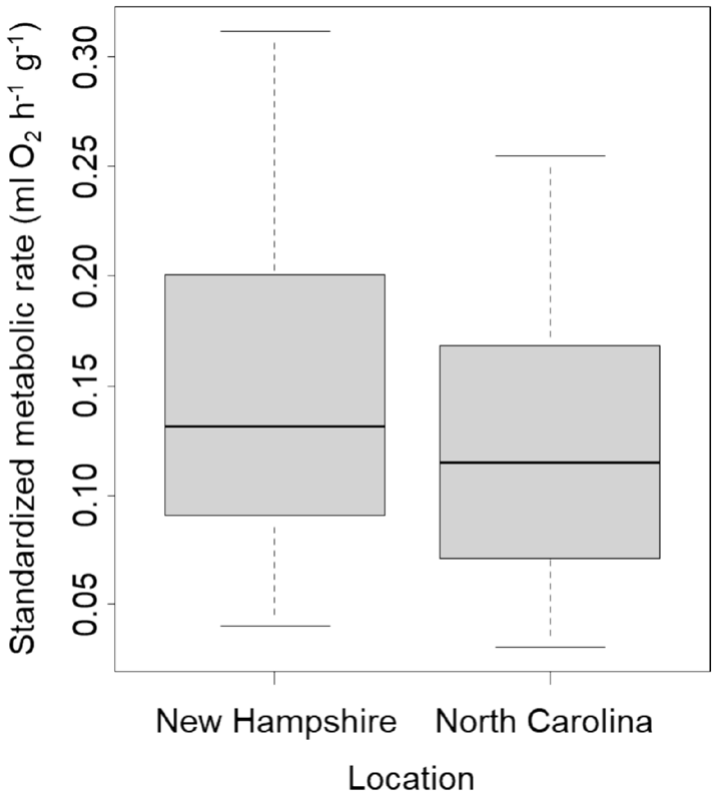
Comparison of aerial metabolic rates at Oregon Inlet, North Carolina (the current southern range limit for *Hemigrapsus sanguineus* ) and previously collected data on the New Hampshire coast.

We found no significant difference between the metabolic rate of crabs collected at the southern range limit at Oregon Inlet, North Carolina, and those collected further north in New England (gamma GLM, *t* = −0.219, df = 18, *p* = 0.83). This held true when one outlier from each site was removed from the analysis (linear model, *t* = −1.07, *p* = 0.29, Figure 5).

## 4 Discussion

We encountered *H. sanguineus* somewhat further south than previously documented, though we found no evidence for a reproductive population south of the documented range limit at Oregon Inlet, North Carolina. We additionally show that hard substrates become less available on the open coast to the south of this current range limit, and existing hard substrates are commonly boulders that are embedded in the sandy substrate, differing from the preferred habitat of boulders sitting atop the substrate under which crabs can take refuge (Lohrer, Fukui, et al. 2000). We also showed that there is no substantial difference in the prey community across the range boundary, or in the metabolic expenditures under similar conditions for crabs at the range edge compared to further north within the invaded range.

Based on these findings, we suggest that a lack of suitable hard‐substrate habitat (i.e., boulders sitting atop the substrate that provide crevices beneath them where crabs can hide) limits the further expansion of *H. sanguineus* ' range to the south. However, while the lack of suitable habitat may be the ultimate driver, this is potentially linked to multiple ancillary contributing factors. First, while acceptable food sources are available on hard‐substrate habitats to the south of the current range limit, these foods are found primarily on exposed vertical surfaces of boulders, limiting the ability of crabs to simultaneously eat and take refuge under boulders. Second, given the similarity in metabolic patterns throughout the range, elevated temperatures toward the south should increase overall energetic costs, especially in the absence of relatively thick boulders sitting atop the substrate that can provide thermal refuge. This in turn could potentially leave less energy for growth and reproduction. This is consistent with smaller clutch sizes observed at the southern range edge compared to the rest of the range (Griffen et al. 2024). In addition to overall warmer temperatures towards the south, thermal maxima during daytime low tides are likely highest at the southern end of the range, and warm temperature extremes in the absence of thermal refuges may therefore also be a factor establishing the southern range limit. Third, *H. sanguineus* is readily consumed by predatory fish (Heinonen and Auster 2012), and consumption by native consumers is known to limit the southward range expansion of the invasive European green crab *Carcinus maenas* along the same coast (deRivera et al. 2005). The lack of suitable refuge habitat south of the current range limit could similarly increase the susceptibility of *H. sanguineus* to predation. Thus, there may be multiple contributing mechanistic drivers that all derive from the lack of suitable substrate.

Adult *H. sanguineus* can tolerate a wide range of salinities, including brief pulses as low as 5 psu (Hudson et al. 2018). Larvae also tolerate a broad range of salinities (as low as 15 psu at 25°C) (Epifanio et al. 1998). This broad tolerance suggests that hard substrates located within bays may further facilitate *H. sanguineus* ' range expansion. In our search for suitable adult habitat, we noted that urban development more commonly occurs up to the shoreline in bays than on the open coast. As a result, there are many more instances of riprap in bays that could provide potential habitat for range expansion.

In the coastal regions of the southeast United States, habitat for *H. sanguineus* is found primarily in anthropogenic structures such as riprap, jetties and sand traps (McDermott 1998). This includes the previously documented southern range limit at Oregon Inlet, North Carolina, as well as our discovery of multiple *H. sanguineus* individuals at the Coast Guard station at the Cape Hatteras ferry terminal. Continued coastal urbanization along the southeast coast that augments the hard substrate through the creation of more anthropogenic structures may therefore facilitate future range expansions of this invader. Other similar examples exist in the region of anthropogenically facilitated range expansions. For example, the barnacle *Megabalanus coccopoma* , introduced from the Pacific, is expanding its range northward with the help of buoys, docks, and towers that serve as settlement sites (Reigel 2015). Similarly, the mangrove tree crab *Aratus pisonii* is expanding its range northward in Florida and Georgia by using boat docks that mimic the vertical structure of mangrove habitats (Cannizzo and Griffen 2019).

As noted by Epifanio (2013), the southern range limit of *H. sanguineus* coincides with Cape Hatteras, North Carolina, a known biogeographic boundary for marine species with larval dispersal. Pappalardo et al. (2015) examined the leakage of species across this biogeographic boundary and found that ~82% of marine invertebrates established on the northern end of Cape Hatteras were also found on the southern side. This level of similarity suggests that Cape Hatteras is fairly permeable to species moving in the equatorial direction. Furthermore, Park et al. (2004) examined the behavior of *H. sanguineus* larvae using a model of export and return and concluded that this species has high dispersal capability. They further drew parallels between the larval behavior of *H. sanguineus* and the widely distributed fiddler crab, *Uca pugnax*, which ranges from Northern Florida to Cape Cod, Massachusetts, crossing the biogeographic boundary at Cape Hatteras. The similar larval biology of these two species therefore suggests that the ability of *H. sanguineus* larvae to cross this boundary may not be the limiting factor in its distribution. Additionally, with climate‐induced changes to the Atlantic meridional overturning circulation, a slowdown in the Gulf Stream is expected (reviewed in Chi et al. 2021), which could further weaken Cape Hatteras as a biogeographic boundary for southward‐moving species that must move counter to the Gulf Stream flow.

In summary, our results suggest that the southern range limit of *H. sanguineus* is likely determined primarily by a lack of suitable hard substrate that provides hiding places for crabs under boulders or other objects. This habitat limitation may interact with other factors we examined, including food that is primarily available on exposed vertical rock surfaces south of the current range limit, and metabolic processes that appear to be similar throughout the invaded range and should therefore lead to increased energy expenditure in warm conditions that lack shade provided by relatively large intertidal habitat refuges. As a result, the southern range limit of *H. sanguineus* has remained constant along the outer banks of North Carolina for more than a quarter century and may be unlikely to spread further south, even with anticipated changes to currents along the coast with continued climate change. However, this optimistic outlook could be altered if continued urbanization of coastal areas includes the creation of more anthropogenic hard substrates south of the current range limit.

## Author Contributions

**Luke Ashworth:** data curation (equal), writing – original draft (equal). **Margo Harris:** investigation (equal), writing – review and editing (equal). **David L. Neu:** investigation (equal), writing – review and editing (equal). **Blaine D. Griffen:** conceptualization (lead), formal analysis (lead), funding acquisition (lead), methodology (lead), project administration (lead), supervision (lead), visualization (lead), writing – review and editing (lead).

## Conflicts of Interest

The authors declare no conflicts of interest.

## Supporting information


Data S1.

## Data Availability

All data used in this study are available as Supporting Information.
